# The novel in vitro reanimation of isolated human and large mammalian heart-lung blocs

**DOI:** 10.1186/s12899-016-0023-2

**Published:** 2016-06-04

**Authors:** Ryan P. Goff, Brian T. Howard, Stephen G. Quallich, Tinen L. Iles, Paul A. Iaizzo

**Affiliations:** Departments of Biomedical Engineering, University of Minnesota, 420 Delaware St. SE, B172 Mayo, Minneapolis, MN 55455 USA; Departments of Surgery, University of Minnesota, 420 Delaware St. SE, B172 Mayo, Minneapolis, MN 55455 USA

**Keywords:** Cardiac anatomy, Cardiac physiology, Heart-lung bloc, Reanimation

## Abstract

**Background:**

In vitro isolated heart preparations are valuable tools for the study of cardiac anatomy and physiology, as well as for preclinical device testing. Such preparations afford investigators a high level of hemodynamic control, independent of host or systemic interactions. Here we hypothesize that recovered human and swine heart-lung blocs can be reanimated using a clear perfusate and elicit viable cardiodynamic and pulmonic function. Further, this approach will facilitate multimodal imaging, which is particularly valuable for the study of both functional anatomy and device-tissue interactions. Five human and 18 swine heart-lung preparations were procured using techniques analogous to those for cardiac transplant. Specimens were then rewarmed and reperfused using modifications of a closed circuit, isolated, beating and ventilated heart-lung preparation. Positive pressure mechanical ventilation was also employed, and epicardial defibrillation was applied to elicit native cardiac sinus rhythm. Videoscopy, fluoroscopy, ultrasound, and infrared imaging were performed for anatomical and experimental study.

**Results:**

Systolic and diastolic left ventricular pressures observed for human and swine specimens were 68/2 ± 11/7 and 74/3 ± 17/5 mmHg, respectively, with associated native heart rates of 80 ± 7 and 96 ± 16 beats per minute. High-resolution imaging within functioning human pulmonary vasculature was obtained among other anatomies of interest. Note that one human specimen elicited poor cardiac performance post defibrillation.

**Conclusions:**

We report the first dynamic videoscopic images of the pulmonary vasculature during viable cardiopulmonary function in isolated reanimated heart-lung blocs. This experimental approach provides unique in vitro opportunities for the study of novel medical therapeutics applied to large mammalian, including human, heart-lung specimens.

**Electronic supplementary material:**

The online version of this article (doi:10.1186/s12899-016-0023-2) contains supplementary material, which is available to authorized users.

## Background

In vitro isolated heart preparations have been a cornerstone of cardiac research since Langendorff’s original methodology was described in the 1890s [1]. The benefits of isolated heart research are numerous and can be remarkable depending on the investigator’s goal. Isolated heart preparations offer a high degree of control over the system including, but not limited to: perfusate selection, flow control, and pre- and after-load variability. For a thorough historic summary of these experimental models the reader is referred to a review by Hill et al. [2]. Furthermore, applied pharmacological studies using such approaches can help elucidate the direct action of agents on isolated cardiac tissues, i.e., while avoiding systemic interactions of other agents or breakdown products (e.g., cardiac-nervous system, hepatic metabolism, etc.) [3].

More recently, high-throughput cardiac perfusion systems are being designed, or now even purchased off the shelf, in which multiple small mammalian hearts can be experimented on simultaneously. Isolated heart preparations have garnered notable insights to mechanisms of arrhythmias [4] and have been reviewed elsewhere [5]. Depending upon the system configuration, a wide range of equipment and modalities are available to the investigator including: electrophysiologic monitoring and pacing, ultrasonography, ultrasonic stimulation, fluoroscopy, infrared thermography, direct visualization via videoscopes, and anatomical mapping systems. Furthermore, the utilization of large mammalian isolated hearts allows for critical preclinical testing of device-tissue interactions in native human anatomies or in large mammalian anatomies highly similar to humans, i.e., if the proper animal model is selected for investigation [6]. Comparative imaging of normal versus pathologic conditions, or interspecies comparisons to determine optimal approaches, models, and designs has been critical for the development of novel therapeutics [7]. To the medical device designer, engineer, or clinician, these insights have proven to be essential for enhancing productivity and are of high educational value [8, 9].

Despite isolated heart preparations being a valuable tool, proper anatomical relationships can be compromised when the lungs are removed. This is particularly true when the pulmonary veins and their native ostia are of interest in the context of therapeutic applications, e.g., for treating atrial fibrillation by the isolation of the pulmonary veins via ablation. More specifically, heart-lung preparations have been utilized previously to elucidate the release of atrial natriuretic peptide [10] and expand the pool of lung transplants to non-beating donors [11]; they have also been used in numerous pharmacologic studies. Interestingly, the first heart-lung preparations are often attributed to Knowlton and Starling [12], however their work acknowledges the methods of Martin [13] which were presented in lecture at Johns Hopkins in 1883. The first publication by Martin of his heart-lung bloc preparation was released in 1881 [14], therefore predating Langendorff’s work by 14 years. In short, this preparation cannulates in situ the superior vena cava and one of the branches coming off the aortic arch. A closed loop is created by which pressure can be monitored, a compliance chamber is incorporated, and pre- and after-loads are varied.

Our laboratory previously reported on the reanimation of human hearts, those non-viable for transplant, using an isolated experimental apparatus [2] and Visible Heart® methodologies [15]. Here we describe expanding these experimental approaches to incorporate intact large mammal heart-lung blocs, including both human and swine specimens. To the authors’ knowledge, this is the first description of large mammalian heart-lung blocks being used to achieve dynamic videoscopic imaging in the pulmonary vasculature. We hypothesized that recovered human and swine heart-lung blocs could be reanimated using a clear perfusate and elicit viable hemodynamic and pulmonic function for numerous hours. Further, this approach would facilitate multimodal imaging, which is particularly valuable for the study of functional anatomy and device-tissue interactions.

## Methods

Human and swine isolated heart-lung blocs were reanimated with either the right, left, or both lungs attached, employing Visible Heart® methodologies [15]. The detailed procurement procedure has been described previously [2, 15]. Briefly, a median sternotomy was performed and an aortic root cannula implanted for delivery of cardioplegia. The inferior vena cava (IVC) was ligated and, just prior to cardioplegia delivery, the IVC for human preparations was typically removed with the liver if it was being recovered for transplant, and the superior vena cava (SVC) and aorta were cross-clamped. Cardioplegia was then delivered under pressure to rapidly cool and arrest the heart. The heart and lungs were then dissected and the heart-lung bloc removed by transection of the major vessels, trachea, and esophagus. The human specimens were then transported on ice to the laboratory within 4–8 h following cross-clamp depending upon the logistics of transportation. The human heart-lung specimens were considered as non-viable for transplantation, i.e., due to unknown cardiac arrest periods, significant cardiac disease, and/or other complications. An analogous procedure was performed on swine hearts in our laboratory (mean animal weight of 84 ± 14 kg; n = 18) using two liters of 4 °C St. Thomas’ cardioplegia for induction of cardiac arrest. We have typically performed these studies with just one lung attached, but the method has been adapted to include both lungs. Preparations with only one lung allow cannulation of the non-utilized pulmonary vein, which may be used to more easily access the left atrium for imaging and/or device placement.

Upon arrival of the human specimens (or after explantation of those from swine), heart-lung blocs were placed in an ice slurry of modified Krebs-Henseleit buffer while cannulation of the great vessels was performed (i.e., IVC, SVC, and aorta). If a one-lung preparation was desired, the left/right pulmonary veins and artery were dissected from the left/right lung, and the lung was removed. These vessels were cannulated as well, and hemostasis valves were fitted for access. If both lungs were desired in the preparation, the pulmonary trunk was cannulated to allow control of the buffer flow, either directing all flow through the lungs or allowing some flow to the reservoir (i.e., a parallel path through the lungs and to the reservoir). An intubation tube was placed in the trachea and connected to a ventilator to control flow through the remaining airways. Preparations were typically ventilated at a respiration rate of 11–15 per minute and at volumes of 150–250 ml per lung.

**Fig. 1:**
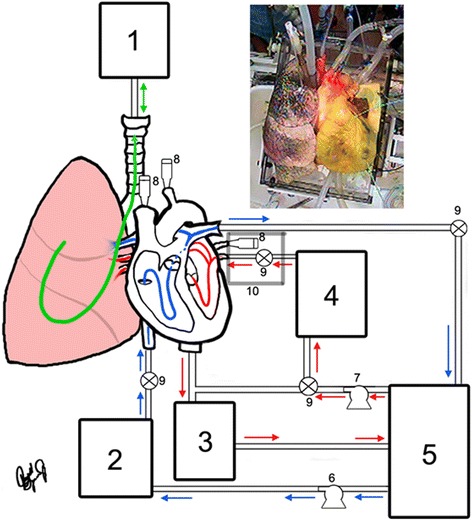
(Top right) External view of human heart 277 in systole and attached to the system. (Center) Flow diagram for a functional heart and lung reanimation consisting of: (1) a respirator connected to the cannulated trachea and thus attached to the lung(s), (2) a pre-load chamber for the right side of the heart, (3) an aortic after-load chamber which mimics the resistance that the left ventricle works against, (4) a left pre-load chamber employed when only one lung is present, (5) an oxygenator reservoir for pooling fluid expelled by any cannulated branch of the pulmonary artery, (6 & 7) fluid pumps to maintain the pre-load pressures, (8) hemostasis valves that allow access for delivery of cameras, instruments, and assorted devices, (9) valves that may also be used to redirect flow as physiologically appropriate, while (10) cannulation of the pulmonary vein(s) are shown here for a right lung preparation, but are absent or translated when either both lungs or the left alone respectively are used

The heart-lung blocs were then connected to the apparatus described in detail previously [15]. A schematic of this system, adapted for heart-lung blocs, can be found in Fig. 1. The system was altered to vary the aforementioned parameters of other isolated heart research systems, and functioned in either partial or four-chamber working mode. Partial working mode is similar to a Langendorff apparatus function, but fluid flow continues through an isolated lung (i.e., the right heart continues to function). The system utilized a cardiovascular bypass oxygenator and heated water jacketed fluid reservoirs to maintain the proper physiologic environment. The preparations were cradled on custom-sized soft foam cushions to support the tissue and minimize alteration to coronary flow. Six to eight liters of modified Krebs-Henseleit buffer were contained within the system, and buffer changes of approximately four liters were performed regularly to wash out residual blood and metabolites, and thus to maintain visualization as desired.

Once the specimen was re-warmed to 37 °C, dobutamine was added to the system and the heart was defibrillated with 34 joules of energy supplied by a programmer-analyzer unit (#88345 Medtronic PLC, Minneapolis, MN, USA) via a pair of external patches (#6721, Medtronic PLC) placed epicardially above and below the ventricles. These hearts generally began beating in native sinus rhythm after a single defibrillation. It should be noted that one human heart developed heart block at two hours post reanimation, and was then paced via a temporary pacing lead at a rate of 60 beats per minute; note that all specimens could be paced if desired. Hemodynamics of the left and right ventricle were recorded by Utah Medical pressure transducers (Model DPT-200, lot#1101991, Midvale, UT, USA) via water columns from venogram balloon tipped catheters (Attain 6215, Medtronic PLC).

High-resolution Olympus commercial endoscopes (Model 1V8200T, Model 1 V8420, Center Valley, PA, USA) were then placed within the hearts and/or lungs to visualize the functional anatomy. To our knowledge, we have been the first group to attempt to visualize the functional anatomy within pulmonary veins and arteries within the lung of functioning human heart-lung blocs.

## Results and Discussion

Using this novel experimental approach, eighteen swine and five human heart-lung blocs were successfully reanimated. Hemodynamic functioning of these in vitro reanimated specimens was typically augmented by the delivery of inotropic agents (1.5 mg dobutamine) and/or by increased dosing with extracellular calcium (0.1 mg/mL, up to ~35–40 mL calcium chloride). Prior to heart recovery, the mean heart rate and blood pressure for the swine were 91 ± 13 beats per minute and 105/56 ± 13/9 mmHg, respectively. Table 1 provides partial cardiac medical histories for the organ donors from which the human hearts were recovered. Table 2 provides the relative early hemodynamic performance data for these reanimated heart-lung preparations, after hemodynamic stabilization post defibrillation. These data points are calculated as 5-min averages at the 1-h time point post defibrillation.

**Table 1:** Summary of donor information and hemodynamic status prior to organ recovery

Human Specimens							
Specimen	Gender	Age (yrs)	Weight (kg)	Cause of Death	HR (bpm)	BP (mmHg)	CVP (mmHg)
HH 277	M	60	113.4	Head trauma	71	105/61	15
HH 284	F	78	54.4	CVA	103	118/70	11
HH 291	F	58	114.7	CVA	92	100/50	12
HH 295	M	34	68.0	Cardiac arrest	92	130/75	-
HH 308	F	36	53.0	CVA, transplant complications	87	97/71	10
Average		53.2	80.7		89.0	110/65	12
Standard Dev.		18.4	31		11.6	14/10	2.2

*BP* blood pressure, *HR* heart rate, *CVA* cerebrovascular accident, *CVP* central venous pressure

**Table 2:** Hemodynamic performance of reanimated heart/lung bloc specimens over 5-min period after hemodynamic stabilization 1-h post defibrillation

Swine Specimens							
Specimen	HR (bpm)	LVSP (mm Hg)	LVEDP (mm Hg)	+dLVP/dt (mm Hg/s)	-dLVP/dt (mm Hg/s)	Tau	Lung
1	95.8	91.2	12.4	982.8	−903.0	31.2	Right
2	91.0	25.0	−2.0	430.8	−343.8	36.2	Right
3	100.0	77.0	−4.0	961.0	−462.0	30.0	Right
4	81.7	73.5	2.3	772.3	−354.5	37.7	Right
5	91.8	85.7	1.3	927.0	−509.8	33.2	Right
6	99.5	62.8	11.3	574.0	−435.0	30.2	Right
7	55.8	82.0	5.7	600.2	−358.2	63.0	Right
8	90.3	79.3	−4.3	842.5	−618.0	33.5	Left
9	92.7	75.7	12.0	623.3	−771.7	32.5	Left
10	102.5	67.5	1.2	637.8	−513.2	29.7	Right
11	76.7	101.7	10.7	786.5	−501.5	39.7	Right
12	123.3	76.3	1.3	729.0	−624.5	26.0	Right
13	124.8	58.7	2.7	762.5	−532.7	24.0	Right
14	84.5	87.2	0.7	888.8	−646.0	54.0	Right
15	114.0	91.7	−2.7	922.3	−808.0	27.5	Right
16	105.0	70.8	0.0	607.7	−446.3	28.8	Right
17	101.3	56.5	3.8	529.8	−317.7	29.8	Right
18	91.2	75.7	7.0	618.3	−810.2	33.0	Right
Average	95.7	74.3	3.3	733.2	−553.1	34.4	
Standard Dev.	16.3	17	5.4	163.6	177.6	9.7	
Human Specimens							
HH 277	85.8	65.7	−7.3	624.5	−475.5	37.2	Right
HH 284	81.2	79.5	1.7	848.2	−377.7	37.5	Right
HH 291	70.3	53.3	8.0	341.3	−273.7	45.3	Both
HH 295	81.3	72.5	4.2	415.7	−343.0	37.7	Right
HH 308	57.2	73.0	0.0	963.0	−469.8	56.2	Right
Average	75.2	68.8	1.3	638.5	−387.9	42.8	
Standard Dev.	11.6	9.9	5.7	268.1	86	8.2	

*HR* heart rate, *LVSP* left ventricular systolic pressure, *LVEDP* left ventricular end-diastolic pressure, *+dLVP/dt* maximal positive derivative of left ventricular pressure with respect to time, −*dLVP/dt* maximal negative derivative of left ventricular pressure with respect to time

It should be noted that one of the early reanimated swine heart-lung specimens (#2) elicited poor hemodynamic performance from the beginning of reanimation. We suspect that injury occurred during isolation and/or that emboli caused poor coronary perfusion. Additionally, recorded data from several hearts elicited negative values for end-diastolic pressures; we suspect that this is due to a vacuum or syphoning effect, potentially occurring in the perfusion system that was modified to incorporate the lungs.

Interestingly, compared to our long-term experience with lone heart reanimations using endoscopes, a large degree of remaining particulate and blood within the lung complicated our initial imaging during certain studies. Therefore, more frequent buffer changes were required to obtain clear, high-fidelity videoscopic images of the functional anatomies.

**Fig. 2:**
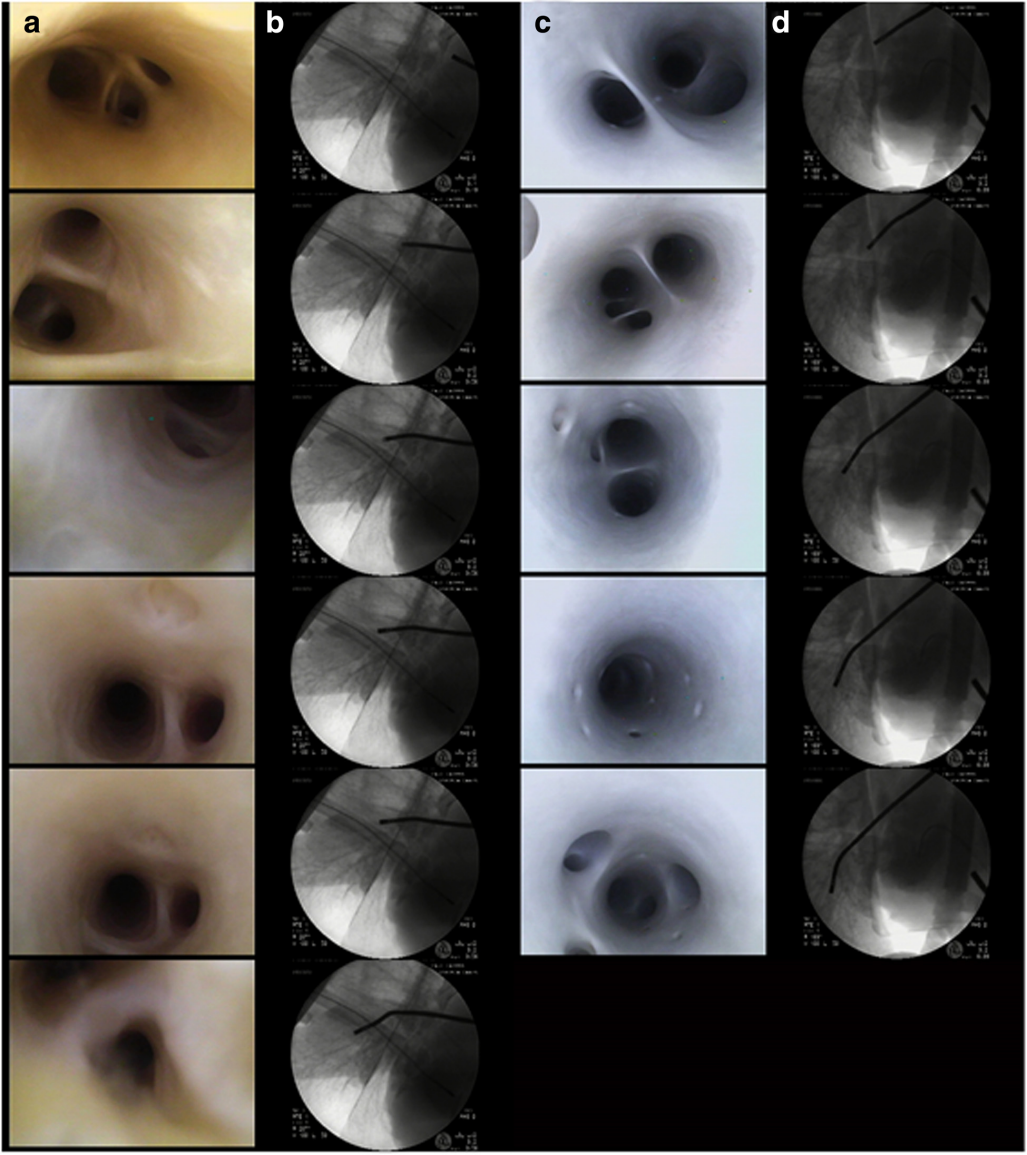
Image series obtained from reanimated human heart-lung bloc 284 (**a**, **b**) and 277 (**c**, **d**). The image series shows the path through the distal pulmonary arteries (**a**, **b**) and veins (**c**, **d**). The corresponding fluoroscopic images (**b**, **d**) in each case show the relative locations of the videoscopes (**a**, **c**). A video of the journey through the vasculature can be viewed as well (see Additional file 1)

As previously mentioned, a primary benefit of this model is the maintenance of proper pulmonary ostia and vessel anatomies. A selected anatomical image series of a videoscope being retracted from either the pulmonary arteries or veins is shown in Fig. 2; a corresponding video can be accessed via supplemental materials (Additional file 1) or online at http://www.vhlab.umn.edu/atlas/left-atrium/pulmonary-vein-distal-pulmonary-branches/index.shtml.

**Fig. 3:**
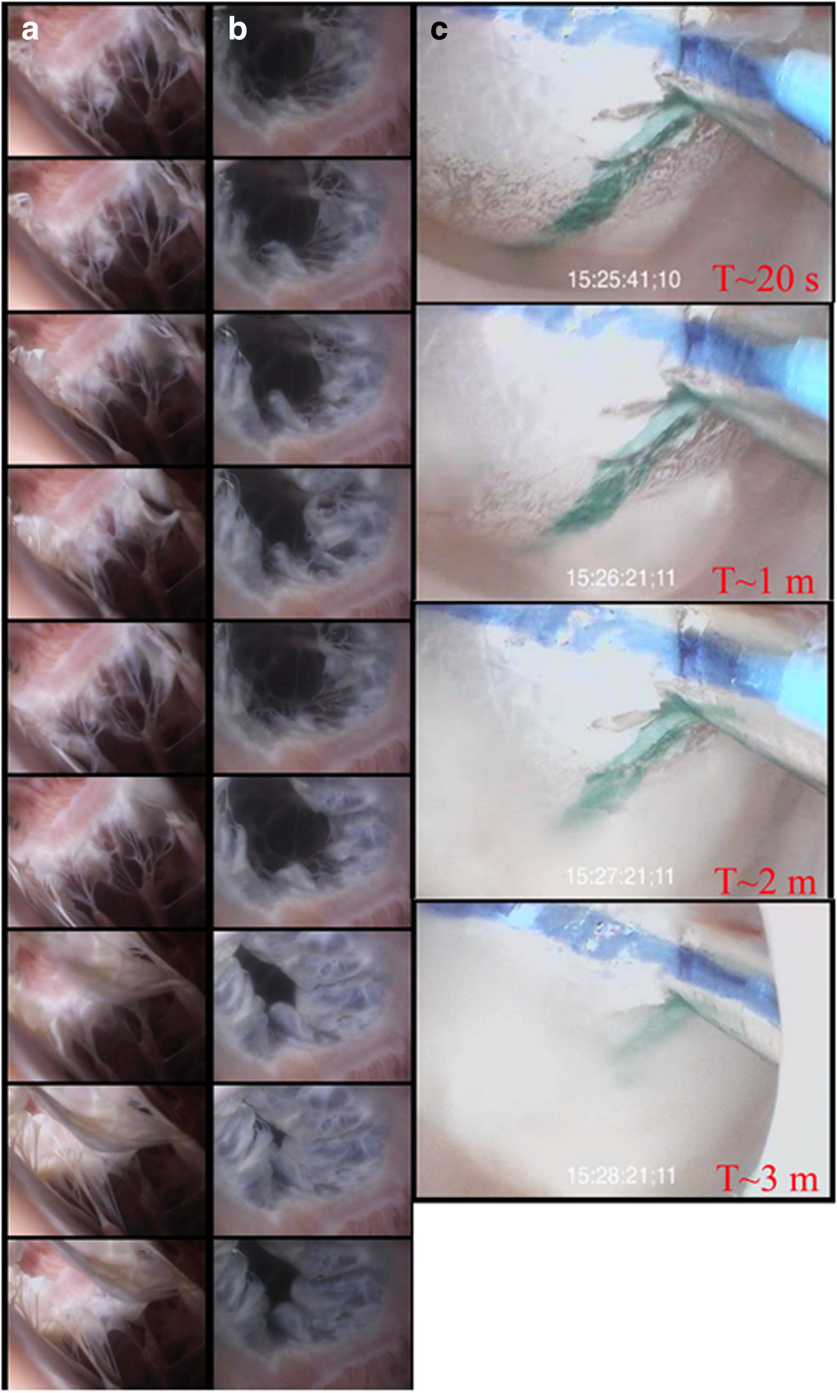
Time series of images from human heart 277. This series shows tricuspid valve closure from the right ventricle (**a**) and right atria (**b**) through slightly greater than one cardiac cycle. In series B, the septal leaflet can be seen toward the bottom left of the images. Images are displayed 1/15th per second apart in time. Panel C displays ice formation on the distal portion of a cryoballoon ablation catheter (Artic Front, Medtronic PLC, Minneapolis, MN, USA) as seen from within the pulmonary vein. The images are spaced 20 s, 1, 2, and 3 min from the start of ablation cooling

In addition to the video, Figs. 2 and 3 both display time series of images captured from these preparations. Figure 2 shows a videoscope being advanced into the pulmonary arteries and veins. It can be seen from the advancement of the videoscope on fluoroscopy (Fig. 2b, d) that vessel caliber decreases with further advancement. Minimal visual differences were noted between obtained human and swine images, with respect to the ability to capture images and the relative image quality. It was noted that the swine pulmonary veins and left atria were typically smaller than those observed within the human specimens. The study of cryoballoon ablation procedures motivated much of the development of this model, and a series of cryoballoon procedures have been performed, as viewed from within the pulmonary vein and displayed in Fig. 3c. This view was obtained by creating a small incision distal to the cryoballoon catheter and advancing the videoscope retrograde. The Additional file 1 of the functioning pulmonary arteries and veins provides the reader with an appreciation for the truly dynamic nature of these vessels, which are usually thought to be relatively passive structures.

The model described here is not without limitations, as is true with all preclinical in vitro systems. Despite supersaturating the buffer with oxygen, there remains a significant difference in the oxygen content of the buffer compared to blood. For this reason, the function of the heart slowly declines over time from the initial reanimation due to ongoing global ischemia. Yet reasonable physiologic function to perform the aforementioned investigations is generally elicited for up to 4 to 8 h, based on our previous experience. Interestingly, we also speculated that the addition of the lungs may possibly extend the functionality of isolated reanimated preparations, a theory that we tend to believe at this point but need more data to substantiate. Most recently, we are employing a full anesthesia suite ventilator to more closely control the ventilation parameters (e.g., provide positive end-expiratory pressure). In such experimentation, one also needs to consider that although it is known that hypothermal transport of organs is protective, global ischemic injury still occurs to some degree. Further, our current system is designed to replicate physiologic pre- and after-loads, but there potentially are important physiological effects that are not completely replicated, such as vessel compliance. Likewise, in our studies the heart-lung blocs are cradled on soft foam sponges which may somewhat focally alter perfusion compared to the natural state; also the pericardium has been removed. Finally, due to the nature of acquiring non-viable donor human heart-lung specimens, there are numerous differences between inherent status of the hearts that cannot be controlled. Such differences in status include, but are not limited to: methods of cardiac arrest, transport times before arriving in the laboratory, clinically required inotropic support, potential air or other emboli in the coronary vasculature (potentially generated during explantation), and/or prior pathologies. Despite being unable to control for these parameters, the described system produced comparable pressures to other isolated human heart alone preparations [2, 16]. As reviewed in Table 2, despite these potential variances, the hemodynamic data are fairly comparable from specimen to specimen. It should be specifically noted that we are extremely grateful and privileged to obtain these donated human heart-lung preparations as gifts for research.

## Conclusions

The experimental techniques described here can be reproducibly employed to study heart-lung bloc preparations with viable cardiac and pulmonary performance. However, it was often observed that cardiac performance (i.e., blood pressure) was somewhat reduced in these preparations compared to their in situ, native behaviors (Tables 1 and 2). This is likely due in part to the hypoxic environment induced by replacing blood with a clear perfusate, but it is also related to the lack of autonomic system innervation. Nevertheless, this novel approach has uniquely enabled visualization of pulmonic anatomies. To the authors’ knowledge, this report has provided first time dynamic videoscopic images of the pulmonary vasculature during self-sustained cardiac function in both reanimated human and swine heart-lung blocs. This unique translational in vitro approach also provides a unique preclinical means to study novel medical therapeutics, i.e., from large mammals, including human and heart-lung specimens.

In a similar embodiment (i.e., without the lungs), this reanimated heart model has been utilized in numerous cardiac studies. In the electrophysiologic area, these studies have included the use of endocardial noncontact mapping, pacemakers, defibrillators, leads, and catheters [17]. The Visible Heart® model has also been employed to study the dynamic nature of valves and transcatheter valve deployment [18]. Importantly these methodological approaches also allow for the simultaneous use of echocardiography and fluoroscopy to guide procedures, such as for comparative imaging [19]. Most recently, this approach has proven to be quite valuable for the study of novel cardiac treatments, such as leadless pacing devices [20]. Yet, the novel addition of a lung(s) to this paradigm allows for any of the prior studies to be conducted, but may in turn reduce the number of hemostasis valve access points that were previously available. Nevertheless, our described reanimation of heart-lung blocs could be adapted to validate swine pulmonic disease models, as swine specimens are more readily available for routine experimentation. It should also be noted that human heart-lung bloc specimens often have associated pulmonary dysfunction. Although our current research has focused on native swine and human specimens, one can imagine the use of this experimental approach for specific disease model testing of either devices or pharmacologic treatments, for instance device or pharmacologic efficacy experiments using COPD or emphysema swine models. In other words, the addition of the lungs to isolated, reanimated heart preparations opens up a wide variety of preclinical/translational cardio-pulmonary research.

Our continued use and enhancement of Visible Heart® methodologies have also facilitated the creation of an open-access educational website, The Atlas of Human Cardiac Anatomy (www.vhlab.umn.edu/atlas) [21]. The anatomical images and videos on this website are free to download and use for presentations and teaching, however we request that proper citations be used. The novel images and/or comparative imaging of functional cardiac anatomies are of high value in teaching the nuances of cardiac anatomy, especially those of active, complex structures such as valve anatomies. This website also provides instructional tutorials on cardiac anatomy and physiology, as well as full cadaveric thoracic cavity dissections. Finally, a cardiac device tutorial is also available, which has been well noted as being beneficial in explaining therapies to patients.

To conclude, this extension of Visible Heart® methodologies to successfully reanimate human and swine heart-lung blocs has enabled novel, functional heart-lung anatomical visualization. Further, unique abilities to image device-tissue interactions using this approach are unparalleled. Therefore, we consider that these images are of high value to medical device designers, educators, and clinicians for both training and educational purposes. The lab will continue to reanimate hearts and heart-lung blocs using these methodologies and thereby enable novel studies of dynamic anatomies, insights into the device-tissue interface, and continued generation of new materials for the free-access Atlas of Human Cardiac Anatomy website.

## Abbreviations

IVC, inferior vena cava; SVC, superior vena cava

## Additional file

Additional file 1: Reanimated Heart-Lung Bloc Imaging. Video obtained from reanimated human heart-lung blocs: 1) human heart 277 (pulmonary veins); 2) human heart 295 (pulmonary arteries); 3) human heart 284 (mitral valve); and 4) human heart 291 (tricuspid valve). (MP4 9135 kb)
